# Correlation between Vascularity and Advancing Histological Grades of Oral Submucous Fibrosis with a Plausible Role in Malignisation: Systematic review of a persisting matter of conflict

**DOI:** 10.18295/squmj.9.2023.057

**Published:** 2024-05-27

**Authors:** Deepak Pandiar, Suvarna K. Nair, Ronell Bologna-Molina, Reshma P. Krishnan, Naina Sivakumar, Rahul Anand, Sahil Chaudhari, Pooja Sharma

**Affiliations:** 1 Department of Oral Pathology and Microbiology, Saveetha Dental College and Hospitals, Saveetha Institute of Medical and Technical Sciences, Saveetha University, Chennai, Tamil Nadu, India; 2 Department in Diagnostics in Oral Pathology and Oral Medicine, University of the Republic, Montevideo, Uruguay; 3 Division of Oral Pathology & Microbiology and Forensic Odontology, CDER, All India Institute Of Medical Sciences, New Delhi, India; 4 Department of Oral Pathology and Microbiology Dr. D.Y. Patil Dental College and Hospital, Dr. D.Y. Patil Vidyapeeth, Sant-Tukaram Nagar, Pimpri, Pune, Maharashtra, India; 5 Department of Conservative Dentistry and Endodontics, Saveetha Dental College and Hospitals, Saveetha Institute of Medical and Technical Sciences, Saveetha University, Chennai, Tamil Nadu, India; 6 Department of Oral and Maxillofacial Pathology, King George's Medical University, Lucknow, India

**Keywords:** Fibrosis, Oral Submucous Fibrosis, Vascularity

## Abstract

**Objectives:**

This study aimed to quantify the vascularity in histological grades of oral submucous fibrosis (OSMF) and to determine if there is any connection between vasculogenesis and malignisation. Recent studies show no significant change in vascularity as the stage advances as opposed to the conventional concept.

**Methods:**

A comprehensive database search until December 2022 was conducted for published articles on vascularity in OSMF following preferred reporting items for systematic reviews and meta-analyses guidelines.

**Results:**

A total of 98 articles were screened of which 13 were included for systematic evaluation. The study included 607 cases, with a definite predilection for the male gender. Of the 13 studies, 11 evaluated mean vascular density. In more than half of the studies, the vascularity decreased as the stage advanced. Similar results were obtained for endothelial cells/μm^2^, mean vascular area percentage and mean vascular area.

**Conclusion:**

The present review supports the prevailing concept that vascularity decreases with the advancement of the OSMF stage. This denies the systemic absorption of carcinogens into the circulation with resultant longer exposure of compromised epithelium and malignisation.

The earliest mention of oral submucous fibrosis (OSMF) most likely dates back to ancient Indian medical literature by ‘Sushruta’, which discusses it as ‘Vidari’ presenting with features such as reduced mouth-opening, pain on eating food and depigmentation of the oral mucosa.^1^ OSMF is usually a habit-related enigmatic, insidious and chronic—yet potentially malignant oral, oropharyngeal and oesophageal—condition seen mainly in natives of Southeast Asian countries, particularly the Indian subcontinent. It is always associated with juxta-epithelial inflammatory reaction followed by progressive stromal fibro-elastic changes such as hyalinisation and homogenisation of collagen bundles, altered vascularity and epithelial atrophy. This results in varied degrees of mucosal stiffness and compromised functional activities.^1^–^3^ OSMF has been estimated to have affected approximately 0.5 million people in the Indian subcontinent, and its highest prevalence is noted to occur in the state of Kerala state in South India. It has also been reported among people of Indian origin across the world.^2^^,^^4^^,^^5^

Vasculature in OSMF has always been a debatable territory with highly variable results from case-control studies.^3^^,^^6^^,^^7^ The prevailing concept is that there is hyperplasia of blood vessels in the very early/early histological grades of OSMF and blood vessels and luminal diameter reduce as the disease progresses.^2^ However, according to a few recent studies, vascularity may remain unaltered or significantly increase as the stage advances, challenging this concept.^6^–^8^ In a morphometric analysis, Rajendran *et al*. demonstrated that mean vascular density does not alter as the stage advances; the luminal diameter and area percentage also showed an increasing trend in their study.^6^ These findings were confirmed immunohistochemically by Desai *et al*. and morphometrically by Fang *et al*.^7^^,^^8^ The varied results are further complicated by variegated methods of assessing vascularity or angiogenesis. While morphometry has been used in some studies on haematoxylin and eosin (H&E)-stained sections, vascularity is assessed by various immunohistochemical markers in other studies. Furthermore, studies have demonstrated that as OSMF turns malignant through dysplastic changes in epithelium, the vascular density increases, depicting a temporal shift in the microenvironment.^3^

Irrespective of all, angiogenesis and vascularity are indeed the key factors in the malignant transformation and progression of the disease. As there is a conflict of information in the existing literature regarding vascularity with the advancement of the stage in OSMF, the present systematic review was planned considering the possibility of a connection between vasculogenesis and malignisation. The study aimed to systematically gather and abridge the available data on vascularity and angiogenesis in OSMF to update the current cognisance of the disease progression and malignant transformation.

## Methods

### PROTOCOL AND REGISTRATION

Preferred reporting items for systematic reviews and meta-analyses (PRISMA) guidelines were used to design this systematic review. This review was registered at the International Prospective Register of Systematic Reviews database (code: CRD42021226351). The research question was ‘Does vascularity change with increasing histological grades of oral submucous fibrosis and does it have any correlation with malignant transformation?’ The PICO for the present review is as follows: population - oral submucous fibrosis; intervention - assessment of vascularity in OSMF; comparison - normal healthy controls; and outcome - evaluation of vascularity in histological grades of OSMF and its correlation in malignant transformation.

### ELIGIBILITY CRITERIA

All articles were included in the review if they were (1) full-length original articles published in the English language only and (2) studies including a quantitative assessment of vascularity and/or angiogenesis in OSMF irrespective of the method employed for quantification.

### INFORMATION SOURCES AND SEARCH STRATEGY

Two authors independently searched MEDLINE by PubMed, SCOPUS, Web of Science, EMBASE and Google Scholar for the following keywords alone or in combination: ALL (‘oral submucous fibrosis’/’OSMF’) and ALL (‘vascularity/angiogenesis’, ‘morphometric’, ‘CD31’, ‘CD34’, ‘bFGF’, ‘mast cells’, ‘CD105’, ‘VEGF’, ‘von Willebrand factor’, ‘angiogenic markers’). Articles that ascertained the aforementioned eligibility criteria were included and appraised further to obtain the data.

### SELECTION AND DATA COLLECTION PROCESS

Two researchers individually screened the titles and abstracts of all the articles. The papers that did not meet the eligibility criteria were excluded. The complete articles were read and evaluated for eligibility and the reasons for exclusions were recorded. Any disagreements were resolved by discussions in consensus meetings with other authors. The following information was extracted from the included articles: country of origin, author(s), year of publication, number of cases and controls, histological classification followed and the method used to assess vascularity or angiogenesis. The parameters were mean vascular density (MVD), mean vessel luminal diameter (MVLD), mean vessel area percentage (MVAP), mean vascular perimeter (MVP) and total vascular area (TVA). Briefly, MVD was defined as the mean of the vessel count in the most vascularised areas from 3–5 high-power fields. MVLD and MVP were estimated in a similar way utilising image software—the cursor was used to outline blood vessels at high magnification and the mean was estimated. MVAP signifies evaluation of the area occupied by blood vessels in the entire field and finally, TVA is the total of areas of all traced vessels at ×400 magnification. Additionally, studies involving oral squamous cell carcinoma arising from OSMF were included for comparative evaluation.

### SUMMARY MEASURES

The main outcome was the quantification of vascularity/angiogenesis in histological grades of OSMF.

### DATA SYNTHESIS AND STATISTICAL ANALYSIS

The quantitative data were tabulated and processed in Microsoft Excel (Microsoft Corporation, 2013). Statistical Package for Social Sciences (SPSS), Version 25 (IBM Corp., Armonk, New York, USA) was used to analyse the data.

### RISK OF BIAS ANALYSIS

The Joanna Briggs Institute critical appraisal checklist for analytical cross-sectional studies was used to assess the quality of the included studies where 8 questions were evaluated and answered for various points with ‘Yes’, ‘Not clear’ and ‘No’.^9^ Finally, the studies were categorised into 3 groups: (1) low risk of bias (at least 70% of the quality criteria were fulfilled); (2) moderate risk of bias (between 50–70% of the quality criteria were fulfilled); and (3) high risk of bias (<50% of the quality criteria were fulfilled). Two authors judged the risk of bias in each domain of the tool independently. Any discordance was resolved by a consensus meeting.

## Results

**Figure 1:**
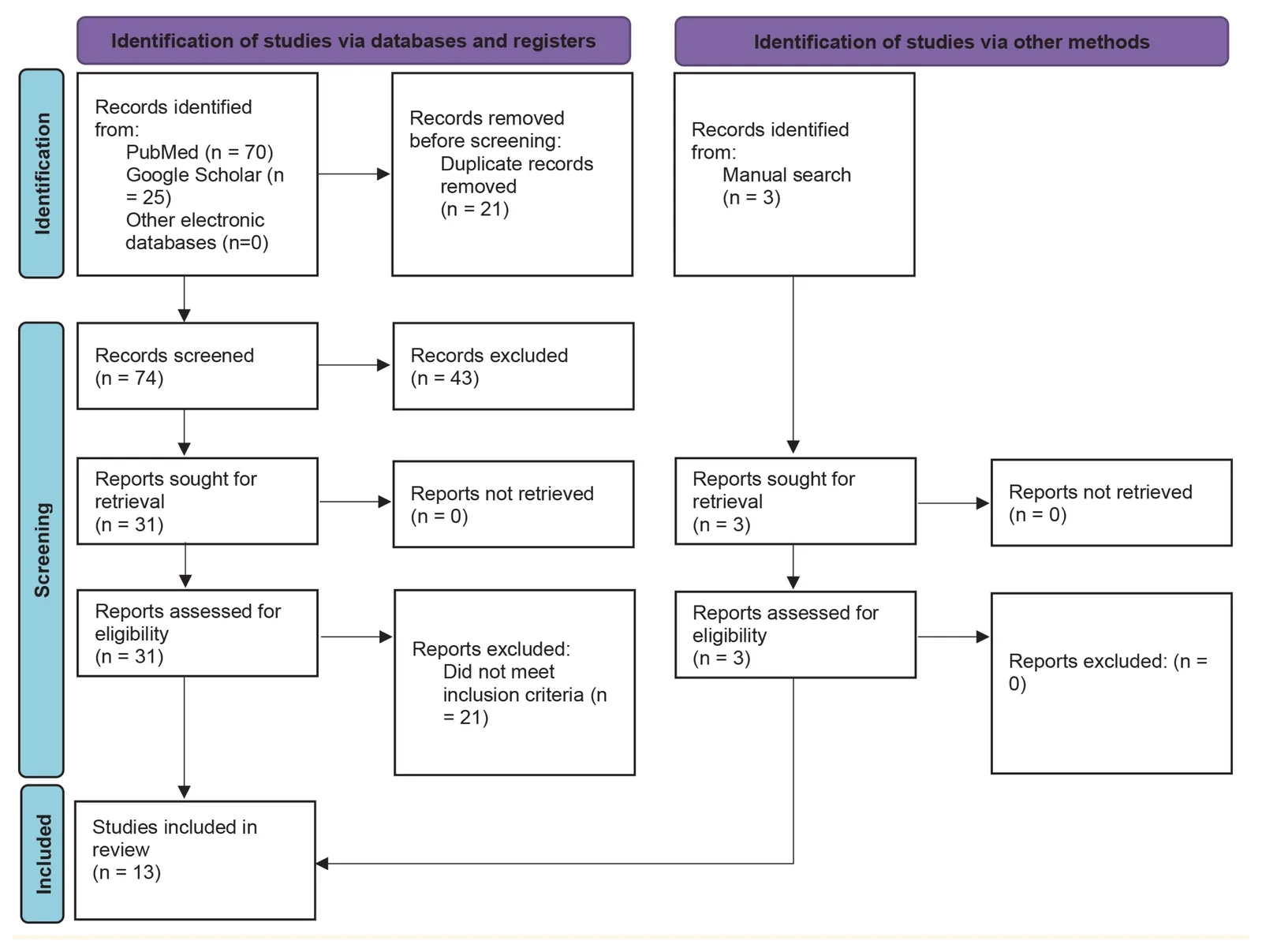
Flowchart showing the study selection process adapted from the preferred reporting items for systematic reviews and meta-analysis 2020.

The search strategy identified 98 articles published until 2022 from various electronic databases. After the removal of 21 duplicate articles, the remaining 77 articles were reviewed by reading the titles and abstract. Of these, 43 articles were excluded with appropriate reasoning. The remaining 34 articles were selected for the eligibility evaluation by reading the full text. At this stage, 21 articles were further excluded due to the lack of quantification of vascularisation in different grades of OSMF. Finally, 13 articles were selected for the present review [Figure 1].^3^^,^^6^^,^^7^^,^^10^–^19^

### CHARACTERISTICS OF THE SELECTED STUDIES

The data extracted from all 13 studies including the details of the country of origin, author(s), number of cases and controls incorporated, classification system followed, methodology used, parameters assessed [Table 1].^3^^,^^6^^,^^7^^,^^10^–^19^

The included studies were conducted in India between 2005 and 2022. A total of 607 OSMF cases and 110 controls were included. In addition, 5 cases of OSMF with dysplasia, 2 OSMFs turning to oral squamous cell carcinoma (OSCC) and 30 OSCC (well differentiated SCC [WDSCC]) were included as comparison groups. Among the selected studies, 53.8% used immunohistochemical markers such as CD34, factor VIII and vascular endothelial growth factor (VEGF) for quantitative assessment of vascularity at varied stages of OSMF. Of the studies, 46.2% used H&E-stained slides for the same. A total of 3 (23.1%) of the studies did not use any control groups,^10^^,^^12^^,^^18^ and only two (15.4%) studies added comparison groups other than control groups.^3^^,^^13^

### DEMOGRAPHIC DATA

The demographic details of cases and controls were retrieved from 8 studies,^3^^,^^11^^,^^12^^,^^14^^,^^15^^,^^17^–^19^ while 5 studies did not provide any such details.^6^^,^^7^^,^^10^^,^^13^^,^^16^ The 13 selected studies included a total sample size of 607 OSMF cases and 110 controls. However, the demographic details were specified only for 368 cases, out of which 285 (77.4%) were males and 83 (22.6%) were females (male:female ratio of 3.44:1). Only 4 of the studies mentioned the history and duration of the habits.^3^^,^^11^^,^^14^^,^^15^

### MEAN VASCULAR DENSITY OF DIFFERENT GRADES OF OSMF

Among the 13 studies, 11 (84.6%) evaluated the MVD in different grades of OSMF.^3^^,^^6^^,^^7^^,^^11^–^17^^,^^19^ Of these 11 studies, 6 (54.5%) reported a decrease in MVD as the grades of OSMF advanced.^3^^,^^12^–^14^^,^^17^^,^^19^ Pandiar and Shameena proposed that MVD reduced from normal mucosa to advanced OSMF and further increased to OSMF with dysplasia and OSMF with OSCC (normal [N] = 40.08) >early ([E] OSMF = 20.48) >moderately advanced ([MA] OSMF = 17.40) >advanced ([A] OSMF = 14.85) <OSMF with dysplasia (22.04) <OSMF = OSCC (OSMF turning malignant = 42.30).^3^ However, 4 other studies showed an increase in MVD from normal mucosa to early OSMF and then decreased to advanced OSMF (N <E >MA >A).^13^^,^^14^^,^^17^^,^^19^ A total of 4 studies failed to establish a statistically significant variation in MVD between different grades of OSMF and the control group.^6^^,^^7^^,^^11^^,^^16^ Out of the 11 studies, 1 showed a discordant data set and was hence categorised separately in this review.^15^

### ENDOTHELIAL CELLS/μM^2^

There were 2 studies that specifically computed the number of endothelial cells/*μ*m^2^ and were thus categorised separately.^10^^,^^18^ Regardless of the parameter used, both articles reported that the number of endothelial cells decreased from very early to advanced OSMF similar to MVD reported in other studies.

### MEAN VASCULAR AREA PERCENTAGE AND MEAN VASCULAR AREA

In total, 7 studies evaluated MVA/MVAP in different grades of OSMF.^6^^,^^7^^,^^10^^,^^11^^,^^13^^,^^18^^,^^19^ There were 4 studies that showed a decrease in MVA/MVAP from early to advanced OSMF.^10^^,^^13^^,^^18^^,^^19^ Murgod *et al*. included WDSCC as a comparison group and demonstrated that MVA/MVAP gradually declined from early to advanced OSMF and further increased to WDSCC.^13^ On the contrary, increased MVAP in advanced OSMF cases when compared to early OSMF was reported by Rajendran *et al*. (control = 0.16; early OSMF = 0.32 and advanced OSMF = 1.02).^6^ A total of 2 studies did not find any significant difference in MVAP between different grades of OSMF.^7^^,^^11^

### MEAN VASCULAR LUMINAL DIAMETER

There were 7 of the 13 studies that evaluated MVLD;^6^^,^^7^^,^^10^^,^^11^^,^^13^^,^^14^^,^^18^ 4 concluded that as the grades of OSMF advanced, the MVLD also reduced.^10^^,^^13^^,^^14^^,^^18^ Among these 4 studies, Nitheash *et al*. reported maximum MVLD in moderately advanced OSMF (2.38 ± 1.10),^14^ but the other 3 studies reported maximum MVLD in early OSMF. Conversely, 1 study group showed an increase in MVLD along with the advancing grades of OSMF, and 2 studies could not put forth any statistically significant difference in MVLD as the advancing grades of OSMF.^6^^,^^7^^,^^11^

### MEAN VASCULAR PERIMETER

Out of the 13 studies, 2 evaluated the MVP and its variability among different grades of OSMF and normal tissue.^11^^,^^14^ Of these 2 studies, 1 proposed a significant reduction of MVP in advanced OSMF when compared to early OSMF (maximum in moderately advanced OSMF) while the other failed to establish any statistically significant variation in different grades of OSMF.^11^^,^^14^

### TOTAL VASCULAR AREA

Only 1 study assessed this parameter and showed that more total vascular area was found in early OSMF when compared to advanced OSMF.^19^ In the studies that used normal tissue samples as comparison groups, all showed an increase in MVD in early OSMF when compared to normal mucosa, except one study that showed higher MVD in normal tissue than that of early OSMF.^3^

### RISK OF BIAS WITHIN THE STUDIES

**Figure 2:**
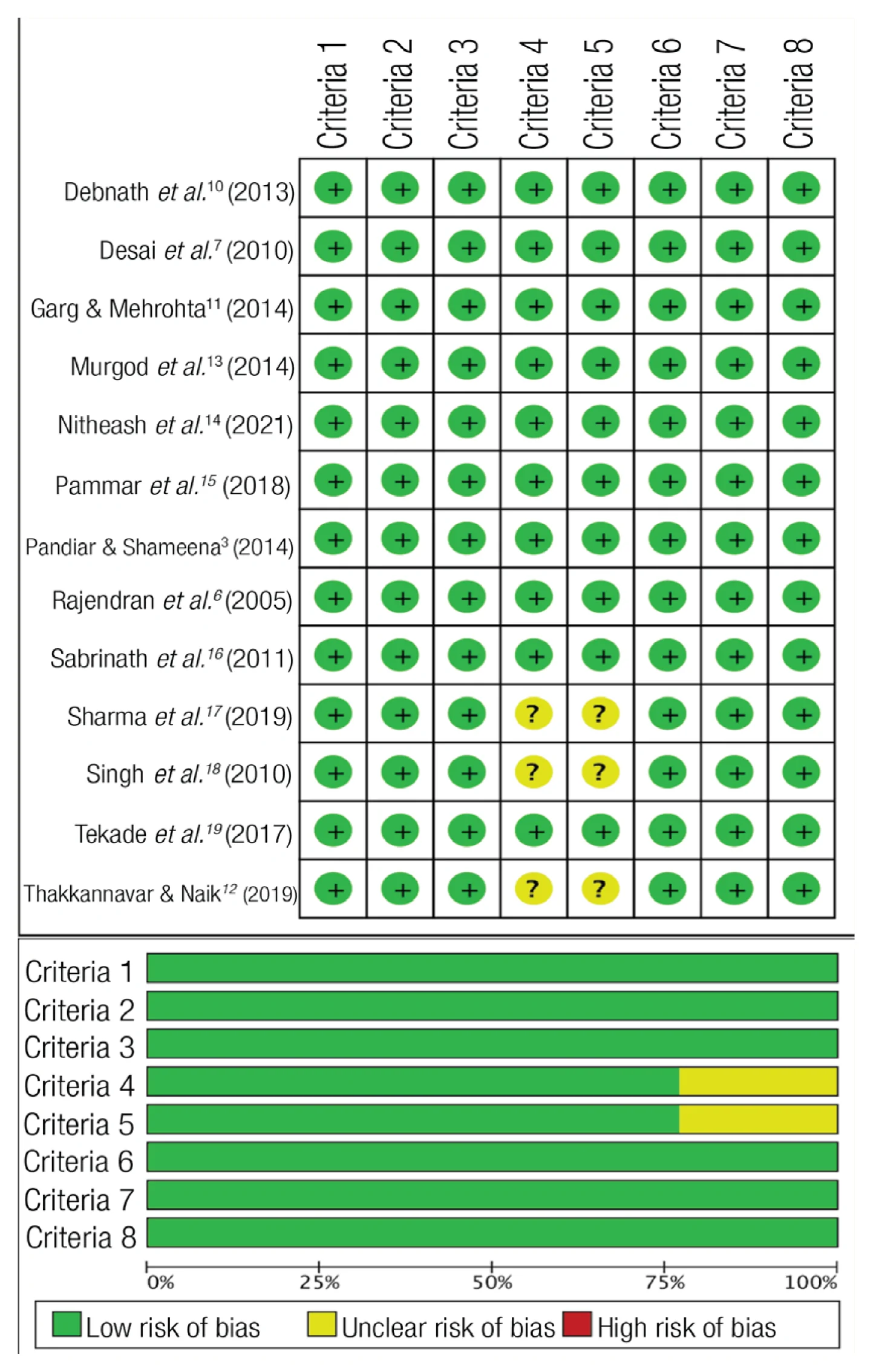
Risk of bias summary and graph (assessed by Joanna Briggs Institute critical appraisal checklist for analytical cross-sectional studies).

Except for 3 studies, all included studies showed a high-quality estimation and a low risk of bias in which unclear risk was estimated in two domains [Figure 2].^12^^,^^17^^,^^18^

## Discussion

OSMF is one of the most common oral diseases in Southeast Asia, especially in the Indian subcontinent, which is potentially malignant. The vascularity of OSMF has always been a conjecture. The vascularity of OSMF varies according to the advancement of grades. According to the conventional concepts, the increased and altered fibroblast proliferation in OSMF results in extensive fibrosis in the connective tissue stroma causing the blood vessels to obliterate. This results in claudication of the vascularity and tissue hypoxia.^20^ However, recent studies challenge the prevailing concept and suggest there is no significant decrease in vascularity with the advancement of OSMF. The present review was orchestrated to shed light on the equivocality of vascularity with the advancement of stages.

The present study confirmed the fact that OSMF is a habit-related progressive disease. Wherever the details were available, the most common habits included areca nut chewing, betel quid with tobacco, paan or commercially available products. It has been previously found that the severity and duration of the habits correlated with increased histopathological grades of OSMF.^21^ In line with the literature, the present review reiterates a preponderance of the male gender.

In the present review, 54.5% of the included studies supported that the MVD decreased with the advancement of OSMF.^3^^,^^12^–^14^^,^^17^^,^^19^ This reinforces the conventional theory that the increase in fibrosis is the result of increased TGF-β-mediated fibroblastic proliferation.^22^^,^^23^ One research group confirmed that arecoline promotes CD147 expression in oral keratinocytes via the TGF-β1 signalling pathway.^22^ The group also opined that CD147 overexpression in OSMF was responsible for the progression of the disease. TGF-β1 appears to play a major role in the fibrotic pathway while cytokine TGF-β2 acts as the contributor.^23^ Areca nut chewing with or without slaked lime through various pathways activates tissue inhibitors of matrix metalloproteinases and induces copper-mediated activation of lysyl oxidases altogether contributing to the increased cross-linking of collagen and further proliferation of fibroblasts. This further increases the fibrosis and results in hyalinisation leading to obliteration of the blood vessels, thus reducing vascularity as the grade advances.^3^ In the present review, 4 studies did not find any statistically significant variation of MVD between the groups of OSMF.^6^^,^^7^^,^^11^^,^^16^ This lack of significant variation could be attributed to hypoxia-induced neovascularisation in advanced OSMF cases. Hypoxia activates HIF-1, which further leads to VEGF mRNA, resulting in angiogenesis.^6^ Another reason for such equivocal results could be the number of samples included in the study, the type of method used for quantification and variation in classification for grading of OSMF. It must be noted that two of these studies used clinical staging.^6^^,^^7^ However, previous studies have found no significant correlation between clinical and histopathological grading explaining the discordance regarding vascularity.^21^^,^^24^^,^^25^

The present systematic review of existing data depicts that the sequence of vascularity with advancing stages of OSMF is mostly consistent with increased angiogenesis in very early and early stages and reduction as the stage advances with a temporal shift in the nature of the inflammatory reaction. The view put forward by Tilakaratne *et al*. holds here that desmoplasia and reduced vascularity of the corium, in the presence of altered cytokine activity, generates a microenvironment for carcinogens of areca nut such as arecoline and arsenic and/or tobacco.^26^ The role of cytokines in fibrosis is well established in other body parts. It has been previously reported that mRNA expression of collagen (I and III) and fibronectin is upregulated in cultured lung fibroblasts through IL-1β and TNF-α.^27^ A few studies have shown contrasting results. However, later research demonstrated that TNF-α inhibited adherence and phagocytosis of collagen.^28^–^30^ The role of these cytokines is also demonstrated in OSMF.^31^–^33^ As the fibrosis increases with a concomitant spatial shift, like the inflammatory reaction and reduced vascularity, an important query arises regarding increased vascularity in OSMF with dysplasia and in the malignant transformation.

In the most recent systematic review and meta-analysis, the malignant transformation rate (MTR) in OSMF has been reported to be 6% with wide heterogeneity among the different nations and ethnic groups.^34^ Indian and Pakistani cohorts showed the highest MTR as compared to the Chinese and Taiwanese populations.^34^ As OSMF is a progressive condition, all the cases should be speculated as a potential candidate for malignisation. Furthermore, most, if not all, cases of transformation have been reported as well differentiated with low incidence of nodal dissemination.^35^^,^^36^ In a recent article, the researchers reported 21 cases of OSCC arising in a background of OSMF and hypothesised a putative role of copper in fibroplasia and vasculogenesis, a phenomenon reported as ‘cuproplasia’.^1^ As the disease advances the fibroblastic activity is stabilised resulting in fibrosis along with collapsed blood vessels explaining the reduced vascularity and decreased systemic absorption of known carcinogens compromising the atrophied epithelium. A few studies have, however, shown no significant change in MVD in the advanced stages with extreme contrasting results from other studies.^6^^,^^7^ As aforementioned, this may be attributed to the methodology, type of assessment tool employed to quantify vasculature and sample size. However, when there is malignant transformation, the role of copper gets reversed and has been hypothesised to be more protective through copper-mediated autophagy, cuproptosis. This opens possibilities for the application of copper in therapeutics in the early stages of OSMF where it bears a role in fibroplasia and vasculogenesis.

## Conclusion

The present review of existing data supports the prevailing concept regarding the vasculature of OSMF that with the advancement of the stage of OSMF, vascularity decreases. This denies the systemic absorption of carcinogens into the circulation with resultant longer exposure of compromised epithelium and malignisation.

## Figures and Tables

**Table 1:** Clinicopathological details and data pertaining to quantitative assessment of vascularity in oral submucous fibrosis cases retrieved from included studies^3^^,^^6^^,^^7^^,^^10^–^19^

Author and year of publication	Country	Classification	Cases	Control	Comparison	Method	Parameter	Statistical test used	Results
Rajendran *et al*.^6^ (2005)	India	Haider *et al*. (2000)	20 Early-8 Advanced-12	10 NOM	Nil	H&E	MVD, MVAP, MVLD	ANOVA	MVD: No significant difference between groups (*P* >0.05) MVAP: Normal < early < advanced (*P* <0.001) MVLD: Normal < early < advanced (*P* <0.01)
Desai *et al*.^7^ (2010)	India	Lai Dr (1995) Clinical	30 Stage 2–4 Stage 3–17 Stage 4–9	10 NOM	Nil	IHC (CD34)	MVD, MVAP, MVLD	ANOVA	MVD: No significant difference between groups (*P* >0.05) MVAP: No significant difference between groups (*P* >0.05) MVLD: No significant difference between groups (*P* >0.05)
Singh *et al.*^18^ (2010)	India	Sirsat and Pindborg (1967)	83 Very Early-9, Early-32, Moderately Advance-39 Advanced-3	None	Nil	H&E Van Gieson’s picric acid, acid fuchsin stain, Masson’s Trichrome	No of endothelial cells/LPF, MVAP, MVLD	Chi-square	1. No of endothelial cells/LPF: Very early > early > moderately advanced > advanced (VE & E: *P* = 0.051) (MA & A: *P* <0.001) 2. MVA: Very early < early > moderately advanced > advanced (VE & E: *P* = 0.051) (MA & A: *P* <0.001) 3. MVLD: Very early < early > moderately advanced > advanced (VE & E: *P* = 0.051) (MA & A: *P* <0.001)
Sabrinath *et al.*^16^ (2011)	India	Sirsat and Pindborg (1967)	30 Very early-9, Early-14, Moderately advanced-7	10 NOM		IHC (Factor VIII)	MVD	ANOVA, independent t-test	1. MVD: Normal < Very early < early < moderately advanced (*P* >0.05 between the groups) 2. MVD: Normal <OSMF (*P* <0.05)
Debnath *et al*.^10^ (2013)	India	Sirsat and Pindborg (1967)	100 Very Early-36, Early-29, Moderately Advanced-28, Advanced-7	None	Nil	H&E	No of Endo cells/sq μm, MVAP, MVLD	ANOVA	1. No of endothelial cells/sq μm: Very early > early > moderately advanced > advanced (*P* <0.001) 2. MVAP: Very early < early > moderately advanced > advanced (*P* <0.001) 3. MVLD: Very early < early > moderately advanced > advanced (*P* <0.001)
Garg and Mehrotra^11^ (2014)	India	Sirsat and Pindborg (1967)	35 Very Early-7, Early-14, Moderately Advanced-9, Advanced-5	10 NOM		H&E	MVAP, MVLD MVP	ANOVA	1. MVAP: No significant difference between groups (*P* = 0.55) 2. MVD: No significant difference between groups (*P* = 0.83) 3. MVP: No significant difference between groups (*P* = 0.90)
Pandiar and Shameena^3^ (2014)	India	Sirsat and Pindborg (1967)	30 Early-11, Moderately Advanced-17, Advanced-2	10 NOM	OSMFdysplasia-5, OSMF-OSCC-2	IHC (CD34)	MVD	ANOVA	1. MVD: Normal > OSMF (*P* <0.001) 2.Normal > early > moderately advanced > advanced (*P* <0.001) 3. Normal > early > moderately advanced > advanced <OSMF-D< OSMF-M (*P* <0.001)
Murgod *et al*.^13^ (2014)	India	Sirsat and Pindborg (1967)	60 30 Early, 30 Advanced	10 NOM	30 WDSCC	H&E	MVD, MVA, MVAP, MVLD		1. MVD: Normal < early > advanced < WDSCC (*P* <0.001) 2. MVA: Normal < early > advanced < WDSCC (*P* <0.001) 3. MVAP: Normal < early > advanced < WDSCC (*P* <0.001) 4. MVLD: Normal < early > advanced < WDSCC (*P* <0.001)
Tekade *et al*.^19^ (2017)	India	Lai Dr (1995) Clinical	45 15-Stage 1, 15-Stage 2, 15-Stage 3	15 NOM	Nil	IHC (CD34)	MVD, MVA, TVA	Kruskal Wallis	1. MVD: Normal < Stage 1 > Stage 2 > Stage 3 (*P* <0.001) 2. MVA: Normal > Stage 1 > Stage 2 > Stage 3 (*P* <0.001) 3. TVA: Normal < Stage 1 > Stage 2 > Stage 3 (*P* <0.001)
Pammar *et al.*^15^ (2018)	India	Lai Dr (1995) Clinical Sirsat and Pindborg (1967)	30 Stage 2–23, Stage 3–6, Stage 4-1 (CLINICAL) Early-3, Moderately advanced-18, Advanced-3	15 NOM	Nil	IHC (CD34 CD105)	MVD	Chi-square	1. MVD: Early > moderately advanced > advanced (*P* value not mentioned) 2. MVD: Normal > OSMF (*P* value not mentioned)
Sharma *et al.*^17^ (2019)	India	Sirsat and Pindborg (1967)	30 Very Early-0, Early-10, Moderately Advanced-10, Advanced-10	10 NOM	Nil	IHC (VEGF, CD34)	MVD	ANOVA, independent t-test	1. MVD: Very early < early > moderately advanced > advanced (*P* <0.001) 2. MVD: Normal < OSMF (*P* <0.001)
Thakkannavar and Naik^12^ (2019)	India	Sirsat and Pindborg (1967)	40 Early-20, Advanced-20	None	Nil	IHC (Factor VIII)	MVD	Fischer’s exact test	1. MVD: Early > advanced (*P* <0.001)
Nitheash *et al.*^14^ (2021)	India	Sirsat and Pindborg (1967)	75 Very Early-0, Early-25, Moderately Advanced-25, Advanced-25	10 NOM	Nil	H&E	MVD, MVLD, MVP	ANOVA	1. MVD: Normal < early > moderately advanced > advanced (*P* <0.05) 2. MVLD: Normal > early < moderately advanced > advanced (*P* <0.05) 3. MVP: Normal < very early < moderately advanced > advanced (*P* <0.05)

NOM = normal oral mucosa; H&E = haematoxylin and eosin; MVD = mean vascular density; MVAP = mean vascular area percentage; MVLD = mean vascular luminal diameter; ANOVA = analysis of variance; IHC = immunohistochemistry; VE = very early; E = early; MA = moderately advanced; A = advanced; MVP = mean vascular perimeter; OSMF = oral submucous fibrosis; OSCC = oral squamous cell carcinoma; WDSCC = well-differentiated squamous cell carcinoma.

## References

[1] Pandiar D, Krishnan RP, Ramani P, Anand R, Sarode S (2023). Oral submucous fibrosis and the malignancy arising from it, could best exemplify the concepts of cuproplasia and cuproptosis. J Stomatol Oral Maxillofac Surg.

[2] Pindborg JJ, Sirsat SM (1966). Oral submucous fibrosis. Oral Surg Oral Med Oral Pathol.

[3] Pandiar D, Shameena P (2014). Immunohistochemical expression of CD34 and basic fibroblast growth factor (bFGF) in oral submucous fibrosis. J Oral Maxillofac Pathol.

[4] Chiu CJ, Chiang CP, Chang ML, Chen HM, Hahn LJ, Hsieh LL (2001). Association between genetic polymorphism of tumor necrosis factor-alpha and risk of oral submucous fibrosis, a pre-cancerous condition of oral cancer. J Dent Res.

[5] Misra SP, Misra V, Dwivedi M, Gupta SC (1998). Oesophageal subepithelial fibrosis: an extension of oral submucosal fibrosis. Postgrad Med J.

[6] Rajendran R, Paul S, Mathews PP, Raghul J, Mohanty M (2005). Characterisation and quantification of mucosal vasculature in oral submucous fibrosis. Indian J Dent Res.

[7] Desai RS, Mamatha GS, Khatri MJ, Shetty SJ (2010). Immunohistochemical expression of CD34 for characterization and quantification of mucosal vasculature and its probable role in malignant transformation of atrophic epithelium in oral submucous fibrosis. Oral Oncol.

[8] Fang CY, Han WN, Fong DY (2000). A morphometric study on the microvessel in oral submucous fibrosis. Hunan Yi Ke Da Xue Xue Bao.

[9] Moola S, Munn Z, Tufanaru C, Aromataris E, Sears K, Sfetcu R Chapter 7: Systematic reviews of etiology and risk. JBI Manual for Evidence Synthesis for analytical cross-sectional studies.

[10] Debnath S, Mitra B, Paul B, Saha TN, Maity A (2013). Morphometric analysis of oral submucous fibrosis and its correlation with histological staging and clinical severity of trismus. Egypt J Ear Nose Throat Allied Sci.

[11] Garg N, Mehrotra RR (2014). Morphometric analysis of epithelial thickness and blood vessels in different grades of oral submucous fibrosis. Malays J Pathol.

[12] Thakkannavar SS, Naik VV (2019). Histochemical and Immunohistochemical Analysis of Collagen Fibers and Microvascular Density in Various Grades of Oral Submucous Fibrosis. Iran J Pathol.

[13] Murgod VV, Kale AD, Angadi PV, Hallikerimath S (2014). Morphometric analysis of the mucosal vasculature in oral submucous fibrosis and its comparison with oral squamous cell carcinoma. J Oral Sci.

[14] Nitheash P, Bastian TS, Cyriac MB, Selvamani M, Malini P (2021). Epithelial and Connective Tissue Changes in Oral Submucous Fibrosis – A Morphometric Analysis. Ann Clin Lab Res.

[15] Pammar C, Nayak RS, Kotrashetti VS, Hosmani J (2018). Comparison of microvessel density using CD34 and CD105 in oral submucous fibrosis and its correlation with clinicopathological features: An immunohistochemical study. J Cancer Res Ther.

[16] Sabarinath B, Sriram G, Saraswathi TR, Sivapathasundharam B (2011). Immunohistochemical evaluation of mast cells and vascular endothelial proliferation in oral submucous fibrosis. Indian J Dent Res.

[17] Sharma E, Tyagi N, Gupta V, Narwal A, Vij H, Lakhnotra D (2019). Role of angiogenesis in oral submucous fibrosis using vascular endothelial growth factor and CD34: An immunohistochemical study. Indian J Dent Res.

[18] Singh M, Chaudhary AK, Pandya S, Debnath S, Singh M, Singh PA (2010). Morphometric analysis in potentially malignant head and neck lesions: Oral submucous fibrosis. Asian Pac J Cancer Prev.

[19] Tekade SA, Chaudhary MS, Tekade SS, Sarode SC, Wanjari SP, Gadbail AR (2017). Early Stage Oral Submucous Fibrosis is Characterized by Increased Vascularity as Opposed to Advanced Stages. J Clin Diagn Res.

[20] Sirsat SM, Pindborg JJ (1967). The vascular response in early and advanced oral submucous fibrosis. Acta Pathol Microbiol Scand.

[21] Pandya S, Chaudhary AK, Singh M, Singh M, Mehrotra R (2009). Correlation of histopathological diagnosis with habits and clinical findings in oral submucous fibrosis. Head Neck Oncol.

[22] Wang W, Xiong H, Hu Z, Zhao R, Hu Y, Chen W (2018). Experimental study on TGF-β1-mediated CD147 expression in oral submucous fibrosis. Oral Dis.

[23] Kamath VV, Krishnamurthy S, Satelur KP, Rajkumar K (2015). Transforming growth factor-β1 and TGF-β2 act synergistically in the fibrotic pathway in oral submucous fibrosis: An immunohistochemical observation. Indian J Med Paediatr Oncol.

[24] Motgi AA, Shete MV, Chavan MS, Diwaan NN, Sapkal R, Channe P (2021). Assessment of correlation between clinical staging, functional staging, and histopathological grading of oral submucous fibrosis. J Carcinog.

[25] Kanneganti S, Kattappagari KK, Tanuja K, Chandra KL, Poosarla C, Baddam VR (2018). Oral submucous fibrosis: Clinical and histopathological correlation of collagen fibers using Masson's trichrome and Van Gieson stains. J NTR Univ Health Sci.

[26] Tilakaratne WM, Klinikowski MF, Saku T, Peters TJ, Warnakulasuriya S (2006). Oral submucous fibrosis: Review on aetiology and pathogenesis. Oral Oncol.

[27] Zhang Y, Lee TC, Guillemin B, Yu MC, Rom WN (1993). Enhanced IL-1-beta and tumour-necrosis-factor-alpha release and messenger-RNA expression in macrophages from idiopathic pulmonary fibrosis or after asbestos exposure. J Immunol.

[28] Dayer JM, de Rochemonteix B, Burrus B, Demczuk S, Dinarello CA (1986). Human recombinant interleukin 1 stimulates collagenase and prostaglandin E2 production by human synovial cells. J Clin Invest.

[29] Mauviel A, Heino J, Kähäri VM, Hartmann DJ, Loyau G, Pujol JP (1991). Comparative effects of interleukin-1 and tumor necrosis factor-alpha on collagen production and corresponding procollagen mRNA levels in human dermal fibroblasts. J Invest Dermatol.

[30] Chou DH, Lee W, McCulloch CA (1996). TNF-alpha inactivation of collagen receptors: implications for fibroblast function and fibrosis. J Immunol.

[31] Haque MF, Meghji S, Khitab U, Harris M (2000). Oral submucous fibrosis patients have altered levels of cytokine production. J Oral Pathol Med.

[32] Haque MF, Harris M, Meghji S, Barrett AW (1998). Immunolocalization of cytokines and growth factors in oral submucous fibrosis. Cytokine.

[33] Kaur J, Jacobs R (2015). Proinflammatory cytokine levels in oral lichen planus, oral leukoplakia, and oral submucous fibrosis. J Korean Assoc Oral Maxillofac Surg.

[34] Murthy V, Mylonas P, Carey B, Yogarajah S, Farnell D, Addison O (2022). Malignant Transformation Rate of Oral Submucous Fibrosis: A Systematic Review and Meta-Analysis. J Clin Med.

[35] Sarode SC, Sarode GS (2013). Better grade of tumor differentiation of oral squamous cell carcinoma arising in background of oral submucous fibrosis. Med Hypotheses.

[36] Chaturvedi P, Vaishampayan SS, Nair S, Nair D, Agarwal JP, Kane SV (2013). Oral squamous cell carcinoma arising in background of oral submucous fibrosis: A clinicopathologically distinct disease. Head Neck.

